# Health care providers’ perspectives on delivering gender equity focused family planning program for young married couples in a cluster randomized controlled trial in rural Maharashtra, India

**DOI:** 10.12688/gatesopenres.13026.1

**Published:** 2019-07-17

**Authors:** Saritha Nair, Anvita Dixit, Mohan Ghule, Madhusudana Battala, Velhal Gajanan, Anindita Dasgupta, Shahina Begum, Sarah Averbach, Balaiah Donta, Jay Silverman, Niranjan Saggurti, Anita Raj

**Affiliations:** 1 ICMR-National Institute of Medical Statistics (NIMS), New Delhi, Delhi, 110029, India; 2 Center on Gender Equity and Health, School of Medicine, University of California San Diego, 9500 Gilman Drive #0507, La Jolla, CA, 92093-0507, USA; 3 Joint Doctoral Program in Public Health (Global Health), University of California San Diego and San Diego State University, San Diego, CA, 92093, USA; 4 Population Council, New Delhi, Delhi, 110003, India; 5 Seth G S Medical College & KEM Hospital, Mumbai, Maharashtra, 400012, India; 6 School of Social Work, Columbia University, New York, NY, 10027, USA; 7 ICMR-National Institute for Research in Reproductive Health, J.M Street, Parel, Mumbai, 400012, India; 8 Department of Obstetrics, Gynecology, and Reproductive Sciences, School of Medicine, University of California San Diego, La Jolla, CA, 92093, USA; 9 Department of Education Studies, Division of Social Sciences, University of California San Diego, San Diego, CA, 92093, USA

**Keywords:** Contraceptive use, family planning, male involvement, gender equity, providers perspectives, India

## Abstract

**Background:** There is increasing programming and research on male engagement and gender-equity (GE) counselling in family planning (FP) services. However, there is a lack of data on healthcare provider’s perspectives on delivering these interventions. The objective of the paper is to present providers’ perspectives on delivering a GE-focused FP intervention, CHARM, to married couples in rural India.

**Methods:**  In-depth interviews were carried out with 22 male village health care providers who were delivering a GE-focused FP intervention, CHARM, to 428 husbands (247 couples) rural Maharashtra, India. Providers were interviewed on their experiences and perspectives during delivery of CHARM. Major domains were identified during a thematic analysis.

**Results:** Local male health providers are interested and can be engaged in delivering a GE-focused FP intervention. Providers believed that the CHARM intervention improves couples’ communication, contraceptive use and strengthened their own capacity to provide FP services in accordance with national FP programmatic efforts. Providers found the low-tech flipchart including pictures and information helpful in supporting their service provision. Providers reported some challenges including lack of privacy and space for counselling, limited access to contraceptive options beyond pill and condom, numerous myths and misconceptions about contraceptives. Providers also reported persistent social norms related to expectancy of pregnancy early in marriage, and son preference.

**Conclusions:** Providers in rural areas with high fertility and related maternal health complications are interested in and can successfully implement a GE-focused FP intervention. Future efforts using this approach may benefit from greater focus to support broader array of spacing contraceptives particularly among first time parents, none or one child parents. There is a need to better support engagement of wives possibly through female provider led sessions parallel to male programs, i.e. gender synchronized rather than couples’ sessions.

**Trial** **registration**: ClinicalTrials.gov NCT01593943, May 8, 2012.

## List of abbreviations

AYUSH: Ayurveda, Yoga, Unani, Siddha and Homeopathy

BAMS: Bachelor of Ayurvedic Medicine and Surgery

BEMS: Bachelor of Electropathic Medicine and Surgery

BHMS: Bachelor of Homeopathic Medicine and Surgery

CHARM: Counseling Husbands to Achieve Reproductive health and Marital equity

DHMS: Diploma in Homoeopathic Medicine and Surgery

FP: Family Planning

FP2020: Family Planning 2020

GE: Gender Equity

ICMR: Indian Council of Medical Research

IEC: Information, Education and Communication

LARC: Long Acting Reversible Contraceptives

MBBS: Bachelor of Medicine and Bachelor of Surgery

MD: Doctor of Medicine

NIRRH: National Institute of Research on Reproductive Health

PHC: Primary Health Center

## Introduction

Globally, more than 220 million women lack access to modern contraceptives ^1^. Of these women, 70 million live in South Asia ^2^. In India, over one-tenth (13%) of women report an unmet need for contraceptives, and rural women are less likely to use contraception ^3, 4^, and more likely to have unwanted pregnancies ^5^. India’s national family planning program has historically promoted permanent sterilization methods, which have failed to address a high rate of unintended pregnancy ^6^. More recent government efforts prioritize an expansion of modern reversible contraceptive options, including more effective long-acting reversible contraceptives (LARCs) ^7, 8^. These approaches fail to engage husbands, who are often key decision-makers in families on issues of fertility and family planning (FP) ^7, 9, 10^. FP providers’ engagement of men in India is important, given research documenting that couple communication, and husband’s support for contraceptive use are key facilitators in married couples’ FP use ^11– 15^.

The FP intervention CHARM (Counseling Husbands to Achieve Reproductive Health and Marital Equity)] engaged male health care providers in India to deliver counseling on FP and gender equity (GE) to couples via engagement of husbands ^16, 17^. Trained providers first implemented FP counseling to husbands in two sessions, and addressed GE concerns related to FP, including son preference, partner violence, respectful couples’ communication and joint decision-making. A third final session was inclusive of both the husband and wife where family planning counseling, couples’ communication, and joint decision-making were discussed, practiced and reinforced. A cluster randomized controlled trial evaluating this intervention found significant increases in contraceptive use and couples’ communication, as well as a reduction of marital sexual violence for CHARM participants compared to a control condition ^17^. These findings are consistent with studies from other national contexts documenting the value of engaging male providers to deliver integrated FP and GE counseling to men ^18– 21^.

To provide further insight into how male providers engage men on issues of family planning and contraceptive use, we analyzed qualitative data from male village health providers delivering the CHARM intervention. Health providers in CHARM were inclusive of both private and public health providers, allopathic (i.e., medical doctor) and AYUSH (Ayurveda, Yoga, Unani, Siddha and Homeopathy i.e., doctors trained in traditional medicine), providing health services in rural communities. This approach is consistent with research recommendations for India, which seek to extend public health services by engaging private and AYUSH providers in FP counseling ^7^. While research examining providers’ perspectives on engagement of men in family planning counseling has been studied in sub-Saharan Africa and the Pacific ^19, 22^, little research engaging male providers has been conducted in the Indian context. This paper examines qualitative data from male village health care providers trained to provide a GE-focused FP intervention, CHARM, to couples in rural Maharashtra, India.

## Methods

### Data source

Data for the study are drawn from in-depth interviews that were part of a cluster randomized control trial to evaluate the impact of the CHARM intervention aimed at increasing utilization of family planning methods and promoting gender equity attitudes among young married couples in rural Maharashtra. In-depth interviews were conducted with the village health care providers after they had completed delivery of the CHARM intervention. This male involvement FP intervention was delivered to 428 husbands (247 couples) over the period of April 2012 to March 2013, and data from providers were collected from August 2013 to September 2013. The project engaged both allopathic (n=9) and non-allopathic (n=13) male health care providers practicing in the selected rural areas where the study was being conducted; all providers who delivered CHARM agreed to and participated in this qualitative study. No financial incentives were given to the providers to participate in the surveys. The study, methodology and CHARM intervention module development are provided in detail elsewhere ^16, 17^. This trial is registered at ClinicalTrials.gov under registration identifier NCT01593943. Questions for which quantitative responses were given are available as *Extended data* ^23^.

### Interviews

In-depth interviews (n=22) were conducted with male providers at their clinics subsequent to completion of intervention delivery for the study. Prior appointment was sought and interviews lasting an average of 30 minutes were conducted after obtaining their informed consent. The interviews collected information on experiences of the providers in delivering intervention, challenges they faced in conducting the sessions, experience with delivery of contraceptives, their perspective of program effectiveness, and their recommendations for program improvement and continuation (see *Extended data* ^23^ for interview tool). 

All interviews were conducted in Marathi or English by trained research staff from the National Institute of Research on Reproductive Health (ICMR-NIRRH), with a Masters-level qualification. Interviewers took notes with direct quotes throughout interviews in the language of the interview, which they expanded upon immediately after each interview was completed in order to record further detail. These files were translated to English, de-identified and sent to project investigators for analysis. Audio recording was not utilized due to providers discomfort with it. All procedures were approved by the Institutional Review Boards of the Indian Council of Medical Research - National Institute for Research in Reproductive Health (D/IECCR/76/2009 and D/ICEC/Sci-01/163/2012, most recent approval on 01/04/2012), University of California San Diego (project #111334, most recent approval on 7/2/2015), and Population Council (Protocol no. 451, most recent approval on 1/15/2014). Written informed consent from participants was obtained by the CHARM Research Team before data collection. All participant data are anonymized.

### Data analysis

Data were analysed using ATLAS.Ti v.7, guided by grounded theory approach where open coding was done and themes were attached to observations during analysis ^24^. Under the supervision of a project co-investigator, two project staff members with master’s degrees in psychology and trained in qualitative analysis independently read the interviews and identified mutually exclusive but possibly linked codes or themes across interviews. Blind coding was done by the coders, where neither was aware of the codes assigned by the other. Additional codes and sub-codes were identified iteratively in this coding process, and reapplied to previous interviews as needed. Coders discussed any coding disagreements; if agreement was unable to be reached, a decision was made by the Co-Investigator who was overseeing the coding process. The four major themes identified were: (1) provider views on how to deliver the CHARM intervention, (2) facilitators and challenges to delivery, (3) perception of intervention effectiveness, and (4) recommendations for the intervention. Quotes best illustrating the themes are presented.

## Results

### Characteristics of health care providers

Providers were, on average 33.6 years old (range 27 to 47) with 3–32 years of experience. Of the 22 providers, 13 had private clinics, and 9 were public providers at government health facilities. There were 13 AYUSH providers with qualifications of Bachelor of Homeopathic Medicine and Surgery (BHMS), Bachelor of Ayurvedic Medicine and Surgery (BAMS), Bachelor of Electropathic Medicine and Surgery (BEMS), or Diploma in Homoeopathic Medicine and Surgery (DHMS). Of the providers, eight were allopathic with Bachelor of Medicine and Bachelor of Surgery (MBBS) and some were currently pursuing their residency Doctor of Medicine (MD) degree. A further eight providers were allopathic with Bachelor of Medicine and Bachelor of Surgery (MBBS) and some were currently pursuing their residency Doctor of Medicine (MD) degree. FP counseling was not part of their routine services; however, they were attending to patients for regular illness. They were trained as part of the study since they had a good rapport with the community around their clinic, high number of clientele, and expressed desire and agreement to be part of the intervention. Raw quantitative results of the survey are available as *Underlying data* ^23^.

### Delivering the CHARM intervention

Providers highlighted the importance of engaging men in family planning and maternal health in the context of a society where men typically make household decisions. They highlighted the importance of utilization of reversible methods of contraception (spacing methods) in addition to sterilization to support healthy birth spacing.

*“It (low contraceptive use and unintended pregnancy) is because of a male dominant culture. We can bring gender equality. Women think that they should use methods but in-laws or husband don’t pay attention to it due to their old thinking or may be because they are not aware about it…if they (couple) adopt spacing then it will be helpful.”* (AYUSH provider, age 32 years)

Providers also discussed the importance of encouraging communication between married couples for jointly deciding the number of children they wished to have, spacing between pregnancies and choice of contraceptives.

*“Many clients have understood that due to 2–3 children and continuous pregnancies many couples get many problems…but they were not getting the right guidance and were avoiding going to government hospital or PHC to take information. We can say that we identified their need and reached them at right time.”* (AYUSH provider, age 32 years)
*“Previously major decisions about family planning were taken with the permission of in-laws but after getting information on family planning methods, men are taking decision jointly by considering their wife’s opinion.”* (AYUSH provider, age 28 years)

They recognized, through the CHARM intervention, the importance of village health providers like themselves for India’s national family planning program, to help improve and sustain contraceptive use among young couples.

*“It is government activity. Till now whatever government has done in the field of family planning or family welfare, it is not successful as per their expectations. So they decided that they will include general practitioners who have reached grassroot level and carry forward CHARM program.”* (AYUSH provider, age 43 years)

They also believed that such expansion would provide opportunity to men and women to freely discuss issues such as spacing of births, son preference, domestic violence, male involvement with these providers.

*“When we were asking in detail about violence, they replied “yes, I physically abuse my wife, but…I rarely slap my wife”. But when I asked them about decision making about contraceptive use, some of them said that they [the couple] took all the decisions though some said that decision about contraceptive use was only his [the man’s] and in-laws*.” (Allopathic provider, age 29 years)
*“-he [the husband of a couple] asked me whether I could do test to confirm the sex of child, or if I could give reference of anyone who can do sonography. I tried to convince him a lot against this, but he said that these things were there in his community, if they got only girls then their community would ban them.”* (AYUSH provider, age 43 years)

At a personal level, health care providers felt that CHARM increased their clientele.

“ *because of this program good relation has developed between me and patients who came for session. They had good follow-up and also started discussing their general illness and illness of family members. Couples, who were not coming to me earlier, also started coming for general treatment.”* (AYUSH provider, age 28 years)

### Facilitators and challenges to intervention delivery

***Facilitators.***

**(a) Interest from participants:** Providers said that they could deliver the programme (the sessions and contents as planned) without much difficulty as recruited participants were interested in receiving the information.

*“One good thing I observed is that people were interested in getting this information, from my four years practice in this area…I was not sure that they will listen to us but when we started giving information, I understood that people feel it is important to listen.”* (AYUSH provider, age 32 years)

**(b) Intervention communication tools:** They also felt that the flipchart provided to them for intervention helped convey messages in an effectively to the participants who had limited ability to read.

*“We gave them basic information, like those who work on vitbhatti [brick kilns] they don’t know what nirodh [condom] is and they have never seen it. We told them and showed them demonstration and flipchart, flipchart is the best thing, flipcharts were very helpful in this program.”* (Allopathic provider, age 29 years)

***Challenges.*** There were two major levels at which barriers to intervention delivery was reported.

**(a) At the participant level**

**(i) Privacy**

Since it was not normative for the health care providers to discuss topics such as contraceptive use and gender equity, they had to conduct sessions according to the comfort level of the participant and the availability of privacy for discussions on contraceptives.

*“They used to feel shy after listening to topics like condom, IUD or menstruation and all* “(Allopathic provider, age 28 years).
“ *Difficulties were from other patients since we have system here that once patient comes he directly gets in doctor’s cabin, and this discussion is so personal that we cannot suddenly open up. Our clinics are not such that there are closed rooms, this disturbance was present, so we had to adapt to it.”* (AYUSH provider, age 32 years)

**(ii) Limited participation of women**

Most providers experienced difficulty in organizing the couple’s session, and consequently participation of women in these sessions was limited. Some providers reported carrying out FP counseling during non-FP health visits by clients or talking to groups of women as well.

*“In the beginning 3-4 husbands came for third session without their wife but later when wife came, then I covered it. Ladies have their own work, ---so it was not possible for them to come. Sometimes when husband or wife got ill at that time they both came and we completed session. But they had not come specially for couple session. Hardly 6-7 couples came for couple session*.” (AYUSH provider, age 32 years)

**(b) At the provider level**

Despite extensive training and careful selection of providers for the intervention, a few providers held biases regarding contraception and gender equity, which affected their intervention delivery. For example, some felt that newly married couples did not need the intervention, as they would not desire contraception due to pronatalist preferences and expectations of these couples. Some also felt that pregnant women were not in need of the intervention since it was not immediately relevant.

*“There were some pregnant women and some just married couples who had desire for child so they should not have been involved, we gave information to them but they didn’t take contraceptive from us.”* (AYUSH provider, age 40 years)

A few providers also felt that promoting reversible methods would not be as useful as sterilization camps to help couples reach their desired contraceptive goals and number of children.

*“I feel that main thing is doing operation [sterilization], whatever we are doing aims at idea that operation should be done on time, so telling verbally or writing down, they will not support us…organize camp and do operations in nearby place*.” (AYUSH provider, age 43 years)
Many providers found it difficult to discuss issues related to marital violence, sometimes due to biased attitudes assuming that it is not an issue.
*“Issues on violence is not that much helpful…those who came to me they don’t have much fights and argument, so I didn’t have to tell them much about it. I used to ask couple whether they had fights or not and they reported that they don’t have fights.”* (AYUSH provider, age 40 years)

### Perception of intervention effectiveness

**(i) Contraceptive knowledge, communication and use**

Participants described the intervention impact on couples’ contraceptive knowledge and use, which corresponds to quantitative findings. In terms of contraceptive knowledge, they felt that their counseling helped clarify how to use contraceptives and myths related to side effects associated with different types of contraceptives.

*“like if we say about condom then 95% people know of condom but no one knew about how to use it. Even among literate people, 80% didn’t know how to use it, after our session they come to know about it*.” (AYUSH provider, age 32 years)
*“They had misconceptions, like someone had told them that it (IUD) got stuck in uterus and had to be operated, or some said that wife told there is problem of itching or there are chances of bleeding, but after giving proper information, even I referred 1–2 patients for IUD insertion.”* (AYUSH provider, age 32 years)

Providers also described that male participation led to the men sharing information about contraceptives with their wives. This helped the couples consider contraceptive use jointly. Male participation also facilitated men being more open to condom use.

*“For some couples, communication has increased. Whatever information was given in second session, they [the husband] had communicated to wife which was seen in third session. When I asked question to wife, she gave answers to it.”* (Allopathic provider, age 27 years)

Improved demand for contraceptives was noted, but providers were not always in the position to meet that demand. Referrals were often given to facilitate uptake from the public facility. But private and AYUSH providers were reported being best suited and able to provide condoms to the participants.

*“People gave very good follow-up, some people used to come repeatedly to ask condom or some used to ask where tablets are available, pills were not available with me so I used to recommend them to go to PHC and take pills from there.”* (AYUSH provider, age 29 years)
*“Yes, people were interested, they also demanded pills or condom, but few had misconceptions about IUD then I explained that there are many advantages of IUD, some also demanded IUD then I referred them to PHC. But preference was given to condom by many respondents than IUD or pills.”* (AYUSH provider, age 42 years).

**(ii) Gender equity issues: marital violence and son preference**

Many providers discussed changes in attitudes of husbands toward their wives, with women indicating being heard and respected more.

*“I had good report from one patient in this context, one respondent’s wife had come to me and said that my husband’s behaviour is better than before, and he is responsive to me now.”* (AYUSH provider, age 47 years)
*“No, they didn’t report [marital violence] but we came to know from neighbours, that there is decrease in violence. Actually there is high rate of alcoholism, after coming home from work they are tired so they drink and if they take more drinks then they beat wife or say bad words*.” (Allopathic provider, age 29 years)

Providers observed that despite intervention efforts to reduce son preference, there had been very little impact on this issue as reported by respondents. The larger social context and family pressures supporting son preference were reported as affecting non-use of contraceptives.

*“In this area, problem is that in-laws pressure couples to have children. They have son preference, if couple wants operation after 2 daughters then they cannot do it. Decision is with in-laws or family members, so I’m satisfied that I explained this information to such people.” (*AYUSH provider, age 42 years)

### Recommendations for the intervention

Overall, providers indicated that they enjoyed delivering the program and felt that the intervention should be sustained. Nonetheless, they had some recommendations to strengthen implementation in future efforts. They recommended that more proximal delivery to clients and booster sessions would be helpful. They also felt more emphasis on son preference was needed based on the inadequate change that occurred for this issue.

*“Instead of bringing each couple one at a time to clinic, we should do this program in their village because bringing these couples to clinic is big challenge, if you gather husband’s in their village and give information then it will be helpful and they will spread this information to other couples who will also get motivated to participate in this program.”* (AYUSH provider, age 32 years)
*“We should do continuous follow up, and emphasize more on son preference, this program is successful in all other issues but this we were not able to emphasize. Health providers and PHC staff should give this information in their program and visits so that there is awareness about this issue.”* (AYUSH provider, age 32 years)

A few mentioned the need for such programmes periodically for couples for retaining the awareness levels of contraceptives and motivate the use among the couples. They also felt that incentives to rural health care providers involved in the programme could motivate them to continue these efforts.

*“These things should be emphasized more. This program should be repeated, if we do survey and again we don’t provide program then there will be no use of whatever done now.”* (AYUSH provider, age 43 years)
*“You should give some incentives to those doctors who participate in this program. This will motivate such providers.*” (AYUSH provider, age 28 years)

## Discussion

Male village health care providers can be engaged in GE and FP intervention to successfully facilitate involvement of men in family planning. The providers reported that participants were responsive to the program, which corresponds with good participation rates reported in the evaluation trial ^17^, and providers indicate that they were able to deliver the program, and the intervention materials such as flipcharts aided in this delivery. The providers’ narratives indicate the value of the male involvement strategy, in improving couple’s communication and family planning outcomes, and in ways that can support the national FP program.

Task shifting has been identified as an effective solution to shortage of healthcare providers, including for family planning services in India ^25, 26^. These findings demonstrate the capacity of both allopathic and AYUSH providers who do not typically focus on family planning to provide family planning interventions. Furthermore, they are able to do this with a GE approach.

While the engagement of these providers shows great promise, important challenges were identified that should be considered for future male engagement intervention program in family planning. Rural providers described difficulty in ensuring a private and comfortable space for open communication for the patients, as well as difficulty being able to provide couples’ counselling due to conflicting schedules between spouses. Parallel sessions for women may be useful. Women’s sessions may benefit from female providers, given the sensitive nature of issues discussed. Provider biases and deeply entrenched norms related to fertility and marriage can make interventions such as CHARM difficult to deliver. Such provider biases have been reported in previous literature in family planning in India and Africa ^27– 29^. Comprehensive training, as well as careful selection of providers is key to effective implementation, and a greater emphasis on person-centered family planning counseling may be useful ^30^. Studies have shown positive effect of training on counseling skills and improved restrictive practices ^31, 32^.

A major challenge in increasing contraceptive use across different types of contraception, including more effective LARCs ^17^, was limited method mix availability. Since the local providers were not trained in certain FP procedures such as IUD insertion or provision of injectable contraceptives, they referred clients to public health facilities for these. Further, the reach of the male CHARM providers was primarily to the male participants and not surprisingly this resulted in distribution often of condoms ^17^, a less effective form of contraceptive relative to LARCs. A gender-synchronized approach, focusing on the values and perspectives of both men and women, and utilizing sex matched providers may improve women’s participation and provision of the full contraceptive method mix available in India is needed. Delivery of the intervention closer to couples’ residence, rather than just the clinic, as recommended by the providers, would also likely support uptake of services. Prior research from India suggests that home visits from community-level health providers may increase use of contraceptives ^33^. Home-based services might be particularly useful but less amenable for FP services that require surgical procedures like IUD insertion, which could be done at a proximally located sub centre.

Findings from this study should be considered in the context of certain study limitations. Providers are from a single intervention conducted for the purposes of a randomized controlled trial, and not in the context of a larger initiative. These study findings may not be generalizable to different settings across India, given that it was limited to a small sample from a single intervention study site in the state of Maharashtra. As interviews were conducted in person by the study team, providers’ responses are subject to both social desirability and recall bias. Finally, interviews were recorded using notes by study staff, but they were not audio recorded, and are therefore susceptible to inaccuracies. Despite these limitations, the study does have a number of strengths. It provides rich and nuanced evidence from providers and corresponds with quantitative findings, offering validity to these. It offers important insights into intervention implementation from providers perspectives, in ways that can help improve future use of the CHARM model and other gender equity focused family planning interventions at community level, especially rural settings.

## Conclusions

This study provides a deeper understanding of implementation of a family planning intervention by the providers and can guide future implementation programs aimed at engaging men in family planning. It shows that the CHARM intervention approach has value, given that providers are interested and responsive, and there were notable effects matching the quantitative findings on contraceptive use, spousal communication, and marital violence. These findings support the value of adding a gender synchronized approach to family planning interventions focused on male engagement, to better engage women and enable provision of a broader array of effective contraceptives.

## Data availability

### Underlying data

openICPSR: CHARM Male Engagement Family Planning Intervention Study Data; Thane District Maharashtra, India, 2012–2014. http://doi.org/10.3886/E100194V4 ^23^.

This project contains the following underlying data:

- CHARM-COUPLES-master-1-12-15_no_created_variablesV3 (complete raw data from the quantitative aspect of the study).
- Copy-of-CHARM-Codebook (codebook used for the dataset).

We cannot provide the qualitative data because even de-identified, these data reveal detailed information on individual participants that can be identifiable (e.g., stories of marriage or family). However, requests for these data can be made by emailing geh@ucsd.edu, and including in the subject line “request for CHARM data." Please include your research question, data analysis plan, and IRB approval with your request.

### Extended data

openICPSR: CHARM Male Engagement Family Planning Intervention Study Data; Thane District Maharashtra, India, 2012–2014. http://doi.org/10.3886/E100194V4 ^23^.

This project contains the following extended data:

- CHARM-baseline-Women-s-survey-with-descriptive-stats (containing questions to and pooled responses from women at baseline)
- CHARM-baseline-Men-s-survey-with-descriptive-stats (containing questions to and pooled responses from men at baseline)
- CHARM-9-month-Women-s-survey-with-descriptive-stats-3-26-14 (containing questions to and pooled responses from women at 9 months).
- CHARM-9-month-Men-s-Survey-with-descriptive-stats-3-26-14(containing questions to and pooled responses from men at 9 months).
- CHARM-Women-18-Month-Survey-with-descriptive-stats (containing questions to and pooled responses from women at 18 months).
- CHARM-Men-18-Month-Survey-with-descriptive-stats (containing questions to and pooled responses from men at 18 months).
- CHARM-IDI-GUIDELINES-VHPs (in-depth interview guidelines)
- CHARM-Variables-Log-1-12-15_no_created_variables (details on variable codes used).

Please note that access to data hosted on openICPSR is unrestricted but will require free registration.

Data are available under the terms of the Creative Commons Attribution 4.0 International license (CC-BY 4.0).
