# Supplementary material for: Health care providers’ perspectives on delivering gender equity focused family planning program for young married couples in a cluster randomized controlled trial in rural Maharashtra, India
Source: Gates Open Res. 2019 Jul 17;3:1508. [Version 1] doi: 10.12688/gatesopenres.13026.1 (PMC7100659; doi:10.12688/gatesopenres.13026.1)
Supplement: Peer review history

# Peer review: Reviewer response for version 1

**DOI:** 10.21956/gatesopenres.14134.r28617

**Published:** 2020-04-01

**Authors:** Arundhati Char

**Affiliations:** 1 U-Respect Foundation, Mumbai, Maharashtra, India

This is an article of importance in its field. The study emphasises the need for Gender Equitable Family Planning programming, keeping men at the helm of the discourse, but not ignoring the need to involve couples in the program. It takes off to corroborate several other researches on male participation or male involvement in family planning programs around the world, basing this study in a cluster randomized controlled trial in India.

 The authors have used in depth interviews among 22 male village health care providers from Aayush and Allopathy systems of medicine; to understand their perspectives with regard to FP counselling, effectiveness and challenges of such interventions, and suggestions for improving reach to men.

 The authors have demonstrated that trained male health care rural providers can be utilised to engage with GE-focused FP counselling, through implementing CHARM. This study was implemented in one small area of rural Maharashtra in India, and corroborates the quantitative survey that preceded this study. So, although this could be termed as a mixed-method study, the results of the qualitative study are presented separately here.

**Abstract:**

- Please open up all abbreviations. For example, please elaborate on CHARM.
- In the background section, your statement "there is a lack of data on healthcare provider’s perspectives …." should read "there is insufficient evidence". There are several examples of provider perspectives in delivering healthcare interventions. One example is Char (2011 ^1^).
- Here in the methods, please clearly describe your study population. For example, male health care providers from both private and public sector...rather than just mentioning ‘providers’.

**Introduction: **                                                

- Further to describing FP2020 and role of men, and Vision 2030 for SRHR, this section can be further strengthened by giving an introduction to the various Indian five-year plans and that ‘male involvement in FP’ was, for the first time, included in the 10 ^th^ five-year plan (2002-2007), and subsequently in the following plans. The 10 ^th^ plan mentioned the need for a paradigm shift from predominantly women-centred programmes to meeting the health care needs of family with emphasis on involvement of men in planned parenthood. The mention here was an outcome of the National Population Policy (2000), that mentioned gender issues impinging on women’s health and the need to encourage male involvement in reproductive health.

This explanation could bring out strongly, the need for the intervention.

**Methods:**

- Overall, the method is well-described and explains the various steps that the research followed. The data analysis is also well-documented.
- The statement “This male involvement FP intervention was delivered to 428 husbands (247 couples)" is a bit confusing to me. Can you clarify this? I wonder if it is …428 husbands including 247 couples, or 428 husbands alone who visited the health centre and 247 couples who did so.?
- Also, it would help if you could describe how many male health care providers did CHARM recruit for the intervention, and what was the selection criteria for their inclusion in the program (even if it was mentioned elsewhere, it would add value repeating it here). This would also give a denominator out of which 22 were selected for the qualitative study.  

**Results:**

- **Characteristics of health care providers:**
   
  - The sentence, “A further eight providers were…degree” is repeated. (Edit)
  - Also, when you say, “some were currently…degree”, should it not be "one was currently…”? Of the 22 providers interviewed, 13 were Ayush practitioners. Of the remaining nine, eight were MBBS, leaving one who was pursuing his MD.
  - Next, were they trained as part of the **study**, or as part of the **CHARM intervention**? Kindly correct this anomaly.

 

- **Delivering the CHARM intervention:**
   
  - I find this title ambiguous and incomplete. Should it be *Importance of delivering CHARM intervention *or *Importance of engaging men through CHARM intervention?*
  - This section focuses on importance of engaging with men on family planning, in a predominantly patriarchal society. However, the next sentence does not tie up to this sub-title. For example, it could be written as, “the providers highlighted the importance of **discussing with men** about the advantages of reversible contraceptive methods to support healthy birth spacing…" Isn’t this what the quote is all about?
  - The next paragraph, “Providers also discussed the importance of encouraging communication between married couples…contraceptives” can 1) be edited to a simple sentence, and 2) the first quote under this segment does not talk of couple communication, but rather communication of clients with the provider. This quote can be moved elsewhere, or the pre-para could be re-worded differently to capture the nuances of both quotes that follow (the second one talks of couple communication).
  - There are quotes that bring out many important issues all in one. It would help to analyse these into different sections rather than wrapping them in a single quote, as told by the respondent. For example, the quote “When we were asking in detail about violence, ….in laws.”, and the next quote talk of domestic violence, contraception decision making and gender preferences. The idea of this section is to bring out the respondent’s experiences of implementing CHARM to men. For me, there is less of nuanced analysis from the qualitative data and more of just quotations.
  - The last quote of this sub-section is good. But could be moved to the next sub-section under **interest from participants. **The fact that providers see an increase in clientele is a facilitator through the intervention and could be linked to the interest levels of the men or couples.
- **Limited participation of women:**
   
  - Reasons for women’s non-participation could be flushed out further and may not be just because they have work. Was data on these reasons, as narrated by the respondent, captured? For example, non-participation could be influenced by timing of session, how and what was communicated to couples as invitation to participate in those FP counselling sessions, comfort of being counselled during a situation where couples (or men) visit the health centre for some other illness, etc.

 

- Overall, throughout the results section, barring a couple of places where conclusions were drawn from more than one response (quotes), most of the important findings seem to emerge from just one respondent having made that particular observation. The qualitative analysis could be more robust. If authors could mention in the text how many providers voiced their opinions or agreed to a particular response, or if there were any outliers for each of the responses, or different opinions or concerns from the respondents, it would make the conclusion or result far more meaningful. Although all findings are very valid and important, drawing conclusions based on just 1 out of 22 respondents does not qualify as a representative view of the provider community.

**Recommendations for the intervention:**

- “More emphasis on son preference was needed…” or rather, more emphasis on discussion around son preference, or, communication on gender equity and equality as a means to counter rigid son preference norms in society was needed. Just a suggestion.

 

**Discussion:**

- A mention of how male participation in family welfare programs can enhance and encourage a gender equitable society would be worth discussing.  Although it has been mentioned that they are able to provide family planning services with a GE approach, the findings do not say so clearly. The fact that very few women participated needs to be discussed in the light of how and what can be tweaked to increase couple participation in such services.
- Also, it would be worthwhile to expand on the discussions of provider perceptions of reaching out to men. For example, male health care providers appreciate the efforts to reach out to men. However, they currently lack the training in doing so. Many times, it’s their perceptions that play a role, which can be brought out in the context of health care providers deciding or perceiving the outcomes of these FP discussions with clients (that such sessions are finally going to lead to encouraging sterilisation, or that couples would be embarrassed to discuss FP in a health care setup due to lack of privacy), which could be the provider’s perception rather than what the couples really ‘feel’ about such discussions. Here the discussion on how provider perceptions play a role on outcomes of such interventions can be highlighted.
- The role of training and adequate support in the form of ‘talking points’ when conducting such sessions with couples should be highlighted. The results state that they found the flipcharts very useful. But it may be that flipcharts were the only tool provided to the hcps. This is not clear from the text. What tools were used and what others can be introduced to scale up or improve outcomes can be further discussed in the context of CHARM, and discussing other such interventions from other literature (for example, Msovela *et al.*, 2016 ^3^).

**Conclusion: **

- The conclusion could draw out some more key take-away's from the study - for example, the preparedness of the health providers to effectively deliver such programs through rigorous training and refresher programs for the providers, that would help reduce their own inhibitions and biases, sufficient support to the service providers by way of specific counselling/educational tools, as well as contraceptive products are very vital. The importance of couple counseling and sensitively incorporating a gendered approach to the family planning intervention would go a long way towards its success.     

**Overall comments:**

- Language edits would make the sentences tighter and more readable.
- The transliteration of quotes from the notes could be better comprehended to make them clearly understood.

I confirm that I have read this submission and believe that I have an appropriate level of expertise to confirm that it is of an acceptable scientific standard, however I have significant reservations, as outlined above.

## References

[1] : Male involvement in Family Planning and Reproductive Health in Rural Central India. *University of Tampere*.2011; Reference source

[2] : National Population Policy. *Government of India*.2000; Reference source

[3] : Implementation and acceptability of strategies instituted for engaging men in family planning services in Kibaha district, Tanzania. *Reprod Health*.2016;13(1) : 10.1186/s12978-016-0253-6 138 10.1186/s12978-016-0253-6 27871299PMC5117686

# Peer review: Reviewer response for version 1

**DOI:** 10.21956/gatesopenres.14134.r28613

**Published:** 2020-03-26

**Authors:** Kate Doyle

**Affiliations:** 1 Promundo-US, Washington, DC, USA

The authors provide an interesting insight into the CHARM intervention, highlighting the feedback from male health care providers who implemented the intervention with men and couples in their communities. The article provides interesting insights, but could be strengthened - most particularly by a) further describing the rationale/approach/definitions of gender equity and male engagement that they employed; b) how this was reflected in the training and materials provided to the health care providers; and c) providing more insights into the opportunities and challenges of such an approach (both in the Results and the Discussion sections). 

 Introduction:

- The authors describe CHARM as a "gender equity" intervention and note that it has been described in greater detail in previous papers. I think it would be good to provide more information in this article for the authors/implementers to describe what their gender equity goals and approach are for CHARM and how this was operationalized within the intervention. It would also be helpful to include a box on the content of the intervention. This would provide the reader with a greater understanding of the depth to which the intervention aimed to promote gender equity.
- Within the paper it is clear that the intervention aimed to engage men as part of its GE approach, but it is not clear if a gender-transformative approach was used - i.e. one that sought to engage men in challenging and transforming inequitable gender norms (that may limit men's involvement in FP, but also hinder women's decision-making and autonomy over their FP choices). Throughout the article, the providers and authors refer to the importance of engaging men in their roles as decision-makers (a more 'instrumentalist' approach to engaging men). Greater description of the GE approach and rationale of the male engagement approach would be helpful to put into context the findings shared. 
- Linked with the above, it appears that the intervention only worked with male health care providers. It would be great to provide more background to this - it can be assumed this is because the implementers believed men would be more easily reached/persuaded by male providers, but this is not explicitly made clear. It would be helpful to know if this was informed by formative research, etc. It is also not clear what proportion of health care providers engaged in FP service provision in the locations of implementation are male - this information would be helpful to understand how the intervention operated within the local context.
- The authors note that "These findings are consistent with studies from other national contexts documenting the value of engaging male providers to deliver integrated FP and GE counseling to men." This statement is not factually accurate. Some of the studies included engaged male facilitators - but not health care providers - to deliver gender-transformative programming to men and couples, with the aim of promoting FP and GE. These studies speak more to the importance of gender-responsive or gender-transformative male engagement approaches, rather than the use of male facilitators/providers (which may be but one aspect of why those programs were successful). The statement could be made more factual.
- It would be good to provide more information on what the training for the health care providers entailed particularly from the GE standpoint. Were providers' own gendered attitudes challenged during the training? What supports were provided to the health care providers to discuss violence with their clients?
- I agree with a previous reviewer, that it would be helpful in the second paragraph to provide more background on the CHARM intervention - who is implementing, where, and when. 

Methods:

- In the 'Interviews' section there is a grammatical error in the statement: "Audio recording was not utilized due to providers [note: should be providers'] discomfort with it."

Results:

- As noted by a previous reviewer, some of the quotes 'suggest additional points that would be worth highlighting'. 
- In the 'At the Provider level' section, the authors note that despite extensive training and careful selection of providers, some still had gender biases. As noted above, it would be great to describe in more detail the training and selection process in the introduction.
- In the 'Contraceptive knowledge, communication and use' section, there are some very important reflections on the importance of promoting couple communication and the providers' feedback on this. It would be wonderful to expand upon this and have this be of greater focus in the article. 
- The article highlights some of the challenges that the providers described during their interviews, but not all of these are referenced in the 'Recommendations' section. It would be wonderful to highlight/add any of the suggestions that they had for how to overcome these challenges. 
- In the 'Delivering the CHARM intervention' section there is a grammatical error in the statement: "They also felt that the flipchart provided to them for [note: 'the' is missing] intervention helped convey messages in an [note: remove 'in an'] effectively to the participants who had limited ability to read."
- In the 'Delivering the CHARM intervention' section there is a grammatical error in the statement: "They used to feel shy after listening to topics like condom, IUD, or menstruation and all". [Note: period is missing after all, and the quotations should be reversed.]

Discussion:

- As noted by a previous reviewer, 'There is no mention of any findings related to potentially negative effects on women of the intervention, and it is not clear whether this was because the providers were not asked about this or because providers did not talk about it.' It would strengthen the paper to provide any insights that were gleaned from the providers on this point and to address this in the discussion section. 
- The discussion section could also be strengthened by referring to additional sources (such as those cited by Holmes in her review) and through further reflection similarities/differences in findings from the other male engagement FP approaches the authors cite in the introduction.

I confirm that I have read this submission and believe that I have an appropriate level of expertise to confirm that it is of an acceptable scientific standard.

# Peer review: Reviewer response for version 1

**DOI:** 10.21956/gatesopenres.14134.r28278

**Published:** 2019-12-11

**Authors:** Wendy Holmes

**Affiliations:** 1 Nossal Institute, School of Population and Global Health, University of Melbourne, Melbourne, Vic, Australia

Within a randomized controlled trial of an intervention to increase men’s engagement in family planning the researchers undertook a qualitative study using in-depth interviews to seek the views, knowledge, attitudes and experiences of the male providers of counselling about contraception, and topics related to gender equity such as shared decision-making and partner violence.  

 The authors have made a valuable contribution to efforts to reach international goals to increase use of contraception and to engage men more in sexual, reproductive, and maternal health more generally.

*Introduction:*

- The introduction appropriately begins by noting the high number of women, globally, that lack access to modern contraceptives, but the data referenced is from 2014. More recent data is available reflecting recent progress and the figures given could be updated (United Nations, Department of Economic and Social Affairs, Population Division (2019). *World Contraceptive Use 2019 *(POP/DB/CP/Rev2019).)
- The rationale in the Introduction could be strengthened by referring to the 2030 Agenda for Sustainable Development, target 3.7: "By 2030, ensure universal access to sexual and reproductive health-care services, including for family planning, information and education, and the integration of reproductive health into national strategies and programmes", and to the need to inform the efforts of partnerships that contribute to reaching this target, such as Family Planning 2020 and FIGO (the International Federation of Gynecology and Obstetrics, https://www.figo.org/news/global-unmet-need-modern-contraceptives-0016065).
- In the second paragraph of the Introduction it would be helpful to know who is implementing the CHARM intervention program and when it began. Rural Maharashtra is mentioned in the first para of the Methods section, but I think it is worth also mentioning in the introduction rather than simply “in India”.

 

*Methods section:*

- The descriptions of the conduct of the interviews and the data analysis are good and clear, and the methods used were appropriate. It would be helpful to have a short description of how the question guide was developed. 

 

*Results:*

- There is a useful description of the health care providers, but it is worth mentioning again here that they were all men. This is a relevant fact that is mentioned briefly only once in the Methods section.
- The choice of illustrative quotes is good, but some quotes suggest additional points that would be worth highlighting.
- For example, the quote after “At a personal level, health care providers felt that CHARM increased their clientele” suggests more than this. It suggests that the program enabled good patient-provider relationships and that the counselling acted as an entry point to other health care services. It is worth noting that this is significant because men are less likely than women to have contact with health services, and the increase in chronic diseases makes it important that men feel comfortable to visit a doctor for detection and management.
- It was interesting that a few providers felt that sterilisation camps would be most useful. This suggests that providers’ past experience will influence their attitudes, and that this is worth addressing in such programs.
- Sometimes quotes raise questions that it would be helpful to address, for example, *“Our clinics are not such that there are closed rooms, this disturbance was present, so we had to adapt to it.”* It would be useful to know how they adapted to it. The quote illustrating the point ‘Some also felt that pregnant women were not in need of the intervention since it was not immediately relevant’ raises the question whether counsellors talked with couples about possible future use of contraception.
- The quote under the sub-heading “Limited participation of women” is more about limited participation by couples rather than women.
- There is no mention of any findings related to potentially negative effects on women of the intervention, and it is not clear whether this was because the providers were not asked about this or because providers did not talk about it.  

 

*Discussion:*

- The Discussion section is good but could usefully be expanded.
- I think it is worth mentioning here that involving men in family planning is a useful entry point to increasing their engagement in reproductive and sexual health more generally.
- The potential to inadvertently cause harm by reducing women’s autonomy in care-seeking and decision-making and the need to avoid reinforcing gendered stereotypes of men as the decision-makers should also be discussed (WHO recommendation on interventions to promote the involvement of men during pregnancy, childbirth and after birth (2015). WHO Reproductive Health Library; Geneva: World Health Organization.). The important finding that men’s participation led to sharing information with their wives could be mentioned further in relation to this point. 
- Several points in the findings, such as the influence of family and community in relation to son preference, and the provider’s suggestion that husbands should be given information in their villages, highlighted the need for greater community advocacy and this could be a recommendation in the Discussion. In particular, the need to work with older men and women to change attitudes could be recommended.

 

*References:*

 There is a reasonable range of references, but not very comprehensive. Although this is not a systematic review I think there are benefits to including some more examples of similar work in order to convey the level of interest and significance of engaging men in reproductive and sexual health, including in family planning. For example:

- World Health Organization (2010 ^1^).
- Char (2011 ^2^).
- Sharma *et al.* (2018 ^3^).
- Adelekan *et al.* (2014 ^4^).
- Kura *et al.* (2013 ^5^).
- Kabagenyi *et al.* (2014 ^6^).
- Bloom *et al.* (2000 ^7^).
- Bishwajit *et al.* (2017 ^8^).

 

 Efforts to increase men’s engagement in family planning are also important because it is a valuable entry point to their greater involvement in antenatal and postnatal care, and in the prevention of STIs and HIV:

- Davis *et al.* (2013 ^9^).

- Singh and Ram (2009 ^10^).

 

*Writing:*

- On the whole the paper is well-written but could benefit from some editing for clarity and to reduce some instances of repetition. For example, “Trained providers first implemented FP counselling to husbands in two sessions,” would be better as: “Trained providers first counselled husbands about FP in two sessions.” And “A third final session was inclusive of both the husband and wife…” would be better as “A third final session included husband and wife…”.
- “Gender equity” is an important concept and I think worth writing in full because some readers will not recall what GE stands for. There is also some inconsistency because there are also some uses of “gender equity” in full.
- In the third sentence of the paragraph “Interviews” there is a typo: “The interviews collected information…” rather than “The interviewers…”.

I confirm that I have read this submission and believe that I have an appropriate level of expertise to confirm that it is of an acceptable scientific standard.

## References

[1] : Policy approaches to engaging men and boys in achieving gender equality and health equity. *World Health Organization*.2010; Reference source

[2] : Male Involvement in Family Planning and Reproductive Health in Rural Central India. *University of Tampere*.2011; Reference source

[3] : Factors influencing male participation in reproductive health: a qualitative study. *J Multidiscip Healthc*.2018;11: 10.2147/JMDH.S176267 601-608 10.2147/JMDH.S176267 30425506PMC6203119

[4] : Male Involvement in Family Planning: Challenges and Way Forward. *International Journal of Population Research*.2014;2014: 10.1155/2014/416457 1-9 10.1155/2014/416457

[5] : Male involvement in sexual and reproductive health in the Mendi district, Southern Highlands province of Papua New Guinea: a descriptive study. *Reprod Health*.2013;10: 10.1186/1742-4755-10-46 46 10.1186/1742-4755-10-46 24020945PMC3847125

[6] : Barriers to male involvement in contraceptive uptake and reproductive health services: a qualitative study of men and women's perceptions in two rural districts in Uganda. *Reprod Health*.2014;11(1) : 10.1186/1742-4755-11-21 21 10.1186/1742-4755-11-21 24597502PMC3946591

[7] : What Husbands In Northern India Know About Reproductive Health: Correlates Of Knowledge About Pregnancy And Maternal And Sexual Health. *Journal of Biosocial Science*.2000(32) :237-251 Reference source 10.1017/s002193200000237610765613

[8] : Factors associated with male involvement in reproductive care in Bangladesh. *BMC Public Health*.2017;17(1) : 10.1186/s12889-016-3915-y 10.1186/s12889-016-3915-y 28049458PMC5209938

[9] : Men and maternal and newborn health: benefits, harms, challenges and potential strategies for engaging men. *Burnet Institute, Melbourne: Compass: Women’s and Children’s Health Knowledge Hub*.2013; Reference source

[10] : Men’s Involvement during Pregnancy and Childbirth: Evidence from Rural Ahmadnagar, India. *Population Review*.2009;48(1) : 10.1353/prv.0.0016 10.1353/prv.0.0016

# Peer review: Reviewer response for version 1

**DOI:** 10.21956/gatesopenres.14134.r27800

**Published:** 2019-09-23

**Authors:** Seema Sahay

**Affiliations:** 1 ICMR-National AIDS Research Institute (NARI), Pune, Maharashtra, India

This study delves on gender equity which is an important area of study especially when it is considered in context of male involvement in the family planning issues. The authors have used qualitative research methods and the study highlights the role of health care providers on family planning among young married couples. The authors have been able to show the strengthening of capacities of HCPs to provide family planning services. The study is a linked to a trial. In context of the CHARM intervention, some of the comments/suggestions are as follows: 

1. A brief description of topics covered under CHARM can be added in the methods section. This would assist readers in understanding the context of the findings.
2. Since this is a qualitative study, I see a limitation of using only IDI. There is a need for triangulation: It would add strength if beneficiary data was also added otherwise the results are difficult to hold. It would be prudent to have something (even if it is from a larger study...either refer if published or add the information) to show confirmation of what HCPs are saying. This would add methodological strength to the paper.
3. Men being the key decision makers in India, still data in this paper shows limited participation of women. Only being busy with work cannot be the reason for a woman not accompanying her husband if he asks her. What could be the gaps and was it explored? This needs to be elaborated and presented to show couple's communication issues. It will also inform CHARM intervention for future improvisations.
4. Biases due to provider's views on marriage etc. have been discussed in the discussion section but strong data needs to support this. Results do not augment this.
5. The authors have critically discussed limitations such as surgical procedures/IUD insertion. However, integration of AYUSH for FP is an important recommendation by the authors. But it seems this is concluded based on implementation of CHARM by the AYUSH HCPs. Example: "... *I’m satisfied that I explained this information to such people.”  *What was the response? The authors talk about 'little impact'. This requires more supportive data in terms of both argument and counterargument.  
6. The results are tilted towards AYUSH practitioners. The authors can use allopathic HCP's data to provide reliable findings.

I confirm that I have read this submission and believe that I have an appropriate level of expertise to confirm that it is of an acceptable scientific standard, however I have significant reservations, as outlined above.
